# Impact of biologically effective dose on tremor decrease after stereotactic radiosurgical thalamotomy for essential tremor: a retrospective longitudinal analysis

**DOI:** 10.1007/s10143-024-02296-1

**Published:** 2024-01-31

**Authors:** Constantin Tuleasca, Guillaume Carey, Romain Barriol, Gustavo Touzet, Francois Dubus, Defebvre Luc, Nicolas Carriere, Nicolas Reyns

**Affiliations:** 1 Department of Clinical Neurosciences, Neurosurgery Service and Gamma Knife Center, Lausanne University Hospital (CHUV), Rue du Bugnon 44-46, BH-08, CH-1011 Lausanne, Switzerland; 2 Faculty of Biology and Medicine (FBM), University of Lausanne (UNIL), Lausanne, Switzerland; 3 Ecole Polytechnique Fédérale de Lausanne (EPFL, LTS-5), Lausanne, Switzerland; 4 Univ. Lille, Inserm, CHU Lille, U1172 - LilNCog - Lille Neuroscience & Cognition, Lille, France; 5 Neurosurgery Department, CHU-Lille, Roger Salengro Hospital, 1, Rue Emile Laine, 59000 Lille, France; 6 Medical Physics Department, University Hospital, Lille, France; 7 U1189-ONCO-THAI-Assisted Laser Therapy and Immunotherapy for Oncology, University of Lille, INSERM, CHU-Lille, 59000 Lille, France

**Keywords:** Biologically effective dose, Essential tremor, Radiosurgery, TETRAS

## Abstract

Stereotactic radiosurgery (SRS) is one of the surgical alternatives for drug-resistant essential tremor (ET). Here, we aimed at evaluating whether biologically effective dose (BED_Gy2.47_) is relevant for tremor improvement after stereotactic radiosurgical thalamotomy in a population of patients treated with one (unplugged) isocenter and a uniform dose of 130 Gy. This is a retrospective longitudinal single center study. Seventy-eight consecutive patients were clinically analyzed. Mean age was 69.1 years (median 71, range 36–88). Mean follow-up period was 14 months (median 12, 3–36). Tremor improvement was assessed at 12 months after SRS using the ET rating assessment scale (TETRAS, continuous outcome) and binary (binary outcome). BED was defined for an alpha/beta of 2.47, based upon previous studies considering such a value for the normal brain. Mean BED was 4573.1 Gy_2.47_ (median 4612, 4022.1–4944.7). Mean beam-on time was 64.7 min (median 61.4; 46.8–98.5). There was a statically significant correlation between delta (follow-up minus baseline) in TETRAS (total) with BED (*p* = 0.04; beta coefficient − 0.029) and beam-on time (*p* = 0.03; beta coefficient 0.57) but also between TETRAS (ADL) with BED (*p* = 0.02; beta coefficient 0.038) and beam-on time (*p* = 0.01; beta coefficient 0.71). Fractional polynomial multivariate regression suggested that a BED > 4600 Gy_2.47_ and a beam-on time > 70 min did not further increase clinical efficacy (binary outcome). Adverse radiation events (ARE) were defined as larger MR signature on 1-year follow-up MRI and were present in 7 out of 78 (8.9%) cases, receiving a mean BED of 4650 Gy_2.47_ (median 4650, range 4466–4894). They were clinically relevant with transient hemiparesis in 5 (6.4%) patients, all with BED values higher than 4500 Gy_2.47_. Tremor improvement was correlated with BED Gy_2.47_ after SRS for drug-resistant ET. An optimal BED value for tremor improvement was 4300–4500 Gy_2.47_. ARE appeared for a BED of more than 4500 Gy_2.47_. Such finding should be validated in larger cohorts.

## Introduction

Essential tremor (ET) is one of the most common movement disorders and touches around 1% of the people worldwide [16]. Its incidence expanses with the advancing age, while the age of onset can be as early as childhood. There is an age peak in the second and sixth decades of life [1]. The term “essential” suggests that the source of tremor is currently undiscovered, although several theories with regards to tremor origin exist [25]. Diagnosis is clinical, based upon history taking and clinical examination, suggestive for isolated, two-sided upper-limb action tremor, with or without tremor in other place such as head, larynx (voice tremor), or lower limbs [16]. First line treatment is pharmacological, with propranolol or primidone [46].

Drug-resistant essential tremor can benefit from standard deep-brain stimulation (DBS) [3], high-intensity focused ultrasound (HIFU) [12, 19], or stereotactic radiosurgery (SRS) [26, 35, 37, 45, 46], aiming at the same surgical target, the ventro-intermediate nucleus (VIM). Radiosurgery is classically performed unilaterally, with a dose of 130–150 Gy [22, 24, 26, 29].

Recently, it has been suggested that biologically effective dose (BED) might play a role in various treatment condition outcomes after SRS, more relevant as compared with the classically prescribed physical dose [44]. Previous studies have underlined a key role in pain relief related to trigeminal neuralgia (TN) [38], hearing preservation in the context of vestibular schwannomas (VS) [4, 41, 42], biological cure in secreting pituitary adenomas (PA) [10, 15], or obliteration of arteriovenous malformations (AVM) [28, 39].

Here, we investigate whether BED plays a relevant role in tremor improvement after SRS for ET, performed unilaterally at the level of the VIM, with one and unplugged isocenter and using a uniform dose of 130 Gy.

## Methods

### Study design

We included patients from a single center (Lille University Hospital), which were retrospectively analyzed. The Ethical Committee of Lille University Hospital, France, approved our study (CNIL number 64). Patients provided individual written informed consent for the procedure.

### Patient population

Were evaluated 78 consecutive patients, diagnosed with ET by our movement disorder neurologists. Thalamotomy was performed unilaterally, between February 2015 and January 2021, using the 4C model (Elekta Instruments, AB, Sweden) and ICON (Elekta Instruments, AB, Sweden) starting February 2018. All patients fulfilled the inclusion criteria, with clear diagnosis of ET and no structural abnormalities (such as hippocampal sclerosis, etc.). Data presented in the presented study and pertinent to BED evaluation was initially assessed by a person (CT) not involved in patient’s selection, SRS treatment or follow-up evaluation.

Basic demographic data can be found in Table 1. Mean age at SRS was 69.1 years (median 71, range 36–88). Mean tremor duration was 27.8 years (median 22, range 3–71). The male to female ratio was 48:30.

**Table 1:** Basic demographic data

	Mean (median, range); proportions
Age (years)	69.1 (71; 36–88)
M:F	48:30
Tremor dominance (right: left)	54:24
Tremor duration (years)	27.8 (22; 3–71)
Potential familial tremor (yes: no)	39/78 (50%)
TETRAS (total) Delta (follow-up-baseline)	57.1 (55.2, 34–81.5) − 34.4 (− 38; − 79–29)
TETRAS (ADL) Delta (follow-up-baseline)	30.5 (31, 16–42) − 37.3 (− 35.3; − 93.3–13)
TETRAS (performance) Delta (follow-up-baseline)	27 (26, 14–43.5) − 27.10 (− 30.9; − 69.6–46.8)
Overall clinical improvement (binary)	50/74 (67.6%)
Extended MRI reaction after SRS Of whom with transient motor symptoms	7/78 (8.9%) 5/78 (6.4%)

### Follow-up period

Mean follow-up period was 14 months (median 12, 3–36). Only one patient had a 3-month follow-up and was part of the “binary outcome” analysis.

### Stereotactic radiosurgery technique

In Lille University Hospital, the radio-neurosurgery technique used is Leksell Gamma Knife (LGK) 4C (Elekta Instruments, AB, Sweden) and ICON (from February 2018, onwards). Diffusion tensor imaging (DTI) is acquired without the Leksell type G stereotactic frame (Elekta instruments, AB, Sweden), to avoid artifacts. Moreover, Leksell stereotactic G frame is applied during the treatment day under local anesthesia, followed by a 3D stereotactic volumetric acquisition, including computer tomography (CT) and 1.5 Tesla MRI (T1 without and with contrast enhancement and T2 weighted constructed interference in steady-state (CISS)/Fiesta imaging employing steady-state acquisition (Fiesta) sequences, without contrast enhancement and replacing former ventriculography).

For VIM targeting, the landmarks of interest, such as the anterior and posterior commissure (AC and PC) and the width of the third ventricle, are defined. A single and unplugged 4-mm isocenter is used. Further adjustment of the target is performed based on the position of the internal capsule, without any beam-channel blocking. A uniform physical dose of 130 Gy at the 100% isodose line is prescribed.

### Dosimetric data

Dosimetric data can be found in Table 2. The mean beam-on time was 64.7 min (median 61.4; range 46.8–98.5). The mean radiation dose rate was 2.6 Gy/min (median 2.8; range 1.5–3.6).

**Table 2:** Dosimetric data

	Mean (median, range)
Beam-on time (min)	64.7 (61.4; 46.8–98.5)
Dose (Gy)	130 Gy for all cases
Dose rate (Gy/min)	2.6 (2.8; 1.5–3.6)
BED (Gy 2.47)	4573.1 (4612; 4022.1–4944.7)

### Clinical evaluation and outcome measures

Patients were seen in person every 3 months until 1 year and on yearly basis after. They were evaluated by the same neurology (specialized in movement disorders) and neurosurgery teams.

Clinical evaluation included the following:

- Continuous outcome (*n* = 35): “The Essential Tremor Rating Assessment Scale” (TETRAS) [31, 34] including total, but also activities of daily living (ADL) and performance score [11]; the TETRAS was available for 35 consecutive patients, due to the large territory covered by our institution, with referrals from all over the country.
- Binary outcome (*n* = 78): as evaluated both by the patient and referring neurologist (yes—tremor decreased, no—tremor did not decrease)

Of note, the clinical response at 12 months after SRS was considered, which was sustained at last follow-up in all cases.

### Radiological evaluation

Radiological evaluation comprised brain MRI (including contrast-enhanced in all 78 cases) every 3 months until 1 year and further on yearly basis.

### Definition of adverse radiation events

Adverse radiation events (ARE) were defined radiologically as larger MR signature on 1-year follow-up MRI accompanied by perilesional edema and clinically, as appearance or not of a hemiparesis.

### Primary aim

The primary outcome was to correlate changes in tremor scores (both continuous and binary outcomes) with BED.

### Secondary aim

The secondary outcome was ARE appearance, as well as their potential correlation with BED.

### Biologically effective dose

Biologically effective dose was calculated for an alpha/beta ratio of 2.47 (BED_Gy2.47_), as previously suggested for normal brain and using a methodology originally established by Fowler [13, 14] and further discussed by Barendsen [2] and Hopewell [17]. Couch-in and couch-out, corresponding to complete closure of cobalt-60 sources, were excluded from total time calculation, as no irradiation was performed during this time.

**Fig. 1:**
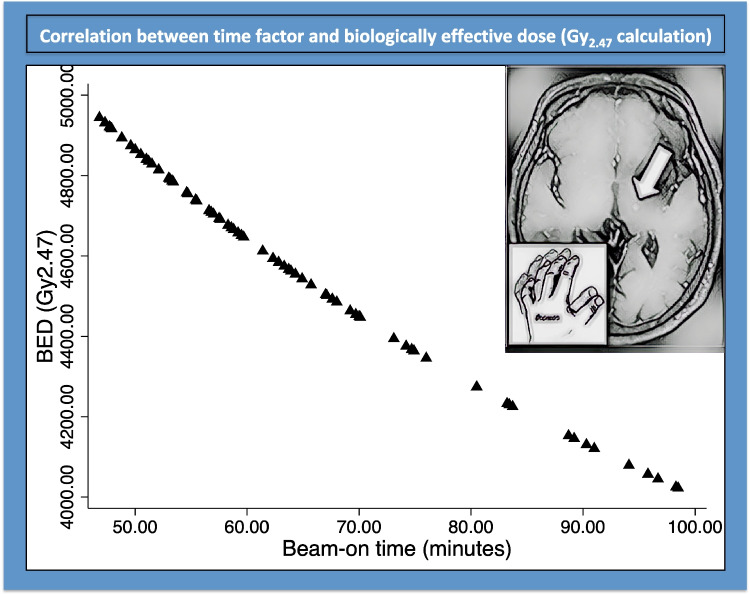
BED as per individual beam-on times (the art piece on the right side of the picture was obtained using artificial intelligence to transform an MRI follow-up picture after stereotactic radiosurgical thalamotomy, depicting the MR signature; deeparteffects.com)

The mean BED was 4573.1 Gy_2.47_ (median 4612; range 4022.1–4944.7). The relationship between the beam-on time and BED can be seen in Fig. 1.

### Statistical analysis

Statistical analysis was performed using Stata 16.1 (StataCorp. 2019, Stata Statistical Software: Release 16. College Station, TX: StataCorp LLC). Descriptive statistics were related as proportion/frequency for categorical data and mean, median, and range for continuous variables. The probability of tremor improvement was assessed using two outcomes. The first (continuous outcome) was to consider a delta of drop in points between follow-up and pretherapeutic examination for the TETRAS score. The second (binary outcome) was to evaluate the tremor improvement as a binary outcome (as defined in the Methods). For the continuous outcome, we used a random-effect linear model, and the strength of the association with covariate was measured using the β coefficient and its calculated *p* value. Fractional polynomial analysis was used to assess for the functional relationship between each predictor and the outcome.

## Results

### Overall tremor improvement in the present series

The mean delta drop in points between the follow-up at 12 months and the initial pretherapeutic value for the TETRAS (total) was − 34.4 (median − 38; range − 79– > 29).

The mean delta drop in points between the follow-up at 12 months and the initial value for the TETRAS (ADL) was − 37.3 (median − 35.3; − 93.3– > 13).

### Probability of tremor score decrease (TETRAS, continuous outcome, delta drop in points between the follow-up at 12 months, and initial pretherapeutic value, as continuous outcome)

Univariate analysis for delta in TETRAS (total) revealed no statistically significant correlation with age (coefficient 0.01; *p* = 0.87), sex (coefficient 0.003, *p* = 0.25), tremor duration (coefficient 0.03, *p* = 0.8), or familial component (coefficient − 0.005, *p* = 0.09).

**Fig. 2:**
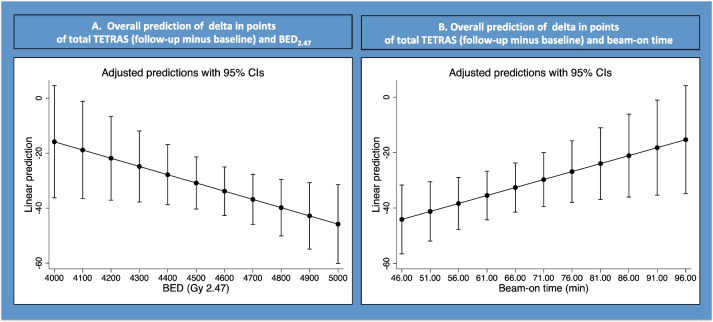
Correlation between the TETRAS (total) and the BED (**A**) and beam-on time (**B**)

Using linear regression, a statistically significant association between tremor improvement, as quantified as the delta drop in points in the TETRAS (total, 12 months follow-up minus baseline) was found with both BED (beta coefficient − 0.029; *p* = 0.04; Table 3; Fig. 2A) and beam-on time (beta coefficient 0.57; *p* = 0.03; Table 3; Fig. 2B).

**Table 3:** Relevant statistical analysis

	Correlation between tremor scores and BED/beam-on time	Coefficient	Standard error	*P* value	95% confidence interval
Delta TETRAS (total)	BED (Gy 2.47)	− 0.029	0.014	**0.04**	− 0.059; − 0.0002
	Beam-on time	0.57	0.25	**0.03**	0.05; 1.09
Delta TETRAS (ADL)	BED (Gy 2.47)	− 0.038	0.015	**0.02**	− 0.07; − 0.005
	Beam-on time	0.711	0.282	**0.01**	0.13; 1.28
Delta TETRAS (performance)	BED (Gy 2.47)	− 0.021	0.015	0.16	− 0.052; 0.009
	Beam-on time	0.413	0.26	0.13	− 0.13; 0.95

Bolded data signifies statistically significant results

Univariate analysis for delta in TETRAS (ADL) revealed no statistically significant correlation with age (coefficient 0.08; *p* = 0.88), sex (coefficient 0.001, *p* = 0.53), tremor duration (coefficient 0.023, *p* = 0.83), or familial component (coefficient − 0.005, *p* = 0.09).

**Fig. 3:**
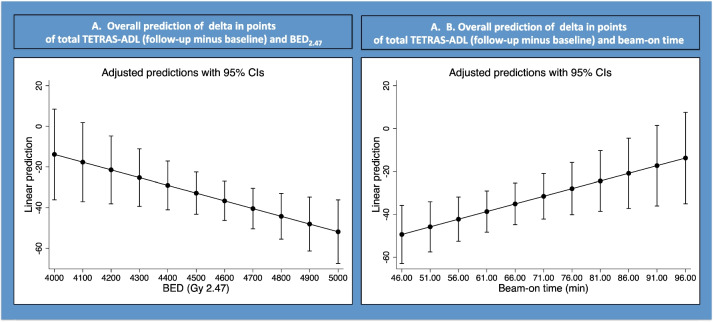
Correlation between the TETRAS (ADL) and the BED (**A**) and beam-on time (**B**)

Using linear regression, a statistically significant association between tremor improvement, as quantified as the delta drop in points in the TETRAS (ADL, 12 months follow-up minus baseline) was found with both BED (beta coefficient − 0.038; *p* = 0.02; Table 3; Fig. 3A) and beam-on time (Fig. 3B).

### Probability of tremor improvement (binary outcome, Fig. 4)

**Fig. 4:**
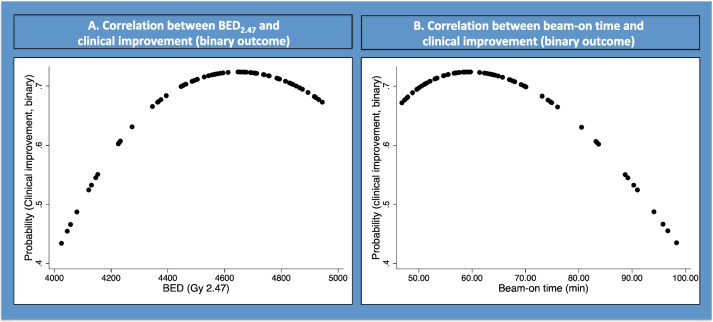
Fractional polynomial showing correlation between clinical improvement (binary) and BED (**A**) and beam-on time (**B**)

Fractional polynomial multivariate regression suggested that a BED > 4600 Gy_2.47_ and a beam-on time > 70 min did not increase clinical efficacy (binary outcome).

Better clinical improvement was obtained for a BED of more than 4350 Gy_2.47_ and a beam-on time of less than 70 min.

Univariate analysis revealed no statistically significant correlation with age (coefficient 2.9; *p* = 0.25), sex (coefficient 0.025, *p* = 0.83), tremor duration (coefficient − 4.8, *p* = 0.32), or familial component (coefficient − 0.04, *p* = 0.72).

### Adverse radiation events

Adverse radiation events (ARE) defined as larger MR signature on 1-year follow-up MRI were present in 7 out of 78 (8.9%) cases, with a mean BED of 4650 Gy_2.47_ (median 4650, range 4466–4894). They were clinically relevant with transient hemiparesis in 5 (6.4%) patients, all with BED values higher than 4500 Gy_2.47_.

## Discussion

In the current study, we assessed whether BED_Gy2.47_ could be relevant for tremor arrest after stereotactic radiosurgical thalamotomy in a series of consecutive patients, treated with a uniform dose of 130 Gy and using no channel blocking. Our data suggest that a better outcome is associated with a BED of more than 4350 Gy_2.47_ and up to 4500 Gy_2.47_, values after which ARE might appear, although their limited number precluded further multivariate analysis.

In a recent systematic review and practice guidelines, it has been suggested that SRS to the unilateral VIM, using a dose between 130 and 150 Gy, is well-tolerated and effective treatment for medically refractory tremor, and one that is recommended by the International Stereotactic Radiosurgery Society [26]. The mean efficacy rates were 88%, and mean clinical complications were 17% (median 2%), the latest being rare and usually transient [26]. In a recent North American study, by Niranjan et al. [29], long-term results are also available, suggestive for 93.2% improvement in tremor, while 60.3% experienced tremor arrest or barely perceptible tremor, with 96% of patients undergoing persistent tremor improvement after a median follow-up of 28 months (range 6–152) [29]. In Europe, the Marseille group evaluated the SRS results for 50 patients in a study by Witjas et al. [45]. The upper limb tremor score improved by 54.2% on the blinded assessment, while all tremor components (rest, postural, and intention) were improved. In the same study, activities of daily living were improved by 72.2% [45]. Side effects were rare and transient in this study [45]. In this respect, our results are in the range of what has been previously reported. Stereotactic radiosurgery remains, in this respect, a safe and valuable treatment option for drug-resistant ET, especially in elderly patients or those with high surgical risk for DBS or radiofrequency thalamotomy [29]. In a recent study, Niranjan et al. [30] also evaluated the safety and efficacy of staged bilateral SRS during a 17-year period for a cohort of 11 patients. Nine of them experienced improvement in at least one Fahn-Tolosa-Marin tremor score [30]. No patient experienced new neurological or radiological adverse effect. Thus, it was concluded that staged SRS is safe and effective in carefully selected patients with bilateral tremor and not eligible for DBS [30].

An open question is whether the Vim targeting procedure, in the context of the SRS purposes, could be performed without the use of contrast-enhanced images. In a recent study, Graciolli Cordeiro et al. [8] evaluated the safety of noncontrast imaging–guided DBS electrode placement in Parkinson’s disease. The authors reviewed 287 cases in which either STN of GPi were targeted [8]. The rate of intracranial hemorrhage was as low as 0.7%, in line with other reported series in the DBS literature [8]. Thus, such an approach could be also considered for SRS purposes. While MR injected images are important for follow-up purposes and the evaluation of MR signature during time after SRS, injected imaging is not as relevant for targeting purposes.

In a recent study by Diaz et al. [9], it was evaluated the individualized anatomy-based targeting for Vim-caudal zona incerta (cZI) DBS in ET. The authors used as reference the intercomissural line (IL), which we also used in our methodology [9]. Their coordinates were located 15 mm lateral to the IL (similar to our approach) or 11 mm lateral to the third ventricle (also similar with our approach), one-fifth the length of the IL posterior to the midcommissural point in the anteroposterior direction, and at the level of the midcommissural point for depth. As a second step and additionally, Diaz et al. [9] targeted the cZI using the most superior T2 axial slice that showed both red nuclei (RNs). A trajectory that passed through the Vim and ended at the cZI was planned [9]. After a follow-up of 31.1 ± 18.4 months, mean tremor improvement rate was 77.9% ± 22.4% and remained stable throughout the follow-up period [9]. We do not routinely target the cZI for SRS thalamotomy purposes. However, we recognize that DBS of the cZI or Vim ± cZI is now a well-acknowledged DBS target for drug-resistant ET [5, 9].

During the past decade and aiming at the same surgical target, HIFU has been suggested as an appealing procedure, producing a controlled thermocoagulation, with an immediate clinical effect [12]. A recent systematic review compared the results of HIFU and SRS for ET [21]. The authors found similar efficacy between the two techniques, with a trend towards higher efficacy yet greater adverse events incidence with HIFU [21]. It was concluded that smaller lesion volumes could mitigate FUS-T off-target effects for greater safety [21]. This is in agreement with a recent statement by Iorio-Morin et al. [19] suggesting that HIFU is being rapidly adopted for the treatment of ET, while the superior popularity of HIFU over SRS appears to rise for reasons other than differences in clinical outcomes. An open question remains whether optimal BED values will potentially enhance even better clinical responses and after shorter intervals after SRS, while decreasing even more the toxicity.

Stereotactic radiosurgery has been commonly considered, and since its invention by the Swedish neurosurgeon Lars Leksell, to be effective enough through a destructive physical mechanism on neural tissue [33]. Particularly, in functional neurosurgery, the desired biological effect can be achieved by targeting a small volume of normal tissue (VIM, trigeminal neuralgia) [23, 35], with a high dose of radiation, or to target a large volume of tissue (epilepsy) with a moderate dose (17–24 Gy at the margin) [27]. Initially, such techniques have been performed based upon the hypothesis that their mechanism of action is purely destructive [33]. However, particularly for SRS of the VIM, recent research suggested not only a local but also a distant effect, with a neuromodulation of brain networks, both structural and functional, particularly visual, in relationship with the clinical effect [6, 7, 40]. Thus, the incisionless, yet considered destructive effect on neural tissue might be insufficient to explain such changes.

Radiobiology of SRS cannot be reasonably considered as an extension of conventional radiotherapy [43]. Up-to-date, current SRS treatment planning has been based on physical dose prescription as a gold standard. However, it is important to assess a more significant biological effect on both the target and the surrounding healthy tissues that the physical dose alone cannot fully explain. In this respect, the time in which a physical dose is delivered is extremely important, particularly for the so-called sublethal effects [14]. Such effects could be depicted using the BED, a concept which had been suggested as relevant for SRS by Hopewell et al. [18]. In a seminal paper, it has been suggested that during SRS for trigeminal neuralgia, safety, and efficacy might be better achieved by prescribing a specific BED instead of a physical dose [38]. This opened the avenue for several other studies, in various pathologies, suggesting higher BED values can be associated with better obliteration rates after SRS for AVMs [28, 39], better biological cures rates after SRS for secreting PA [10, 15], better hearing preservation rates after SRS for VSs [4, 42] or higher rates of tumors decrease after SRS for VSs [36]. Our present study adds to the current evidence suggesting, for the first time, that higher BED values are associated with better tremor arrest rates after SRS for drug-resistant ET.

Previous research suggested that over the course of the 63-month lifespan of the cobalt-60 source, BED decreased annually by 2.2% for TN management, 3.0% for thalamotomy, and 3.5% for capsulotomy, although a clear clinical correlation with BED variations has not been shown [20]. The authors concluded that the use of a new cobalt-60 source after change of an old source considerably increases the predicted BED for functional SRS treatments for the same physical dose prescription [20]. We suggest, based on the current findings, that the BED should be adjusted, rather than changing the cobalt sources, to achieve similar efficacy rates while using old source, while decreasing the toxicity by potentially adjusting the prescribed dose.

## Limitations

Our study has several inherent limitations. The first is related to its retrospective nature, with all bias that such implies. The second is straightforwardly related to the BED formulae. Here, we used a bi-exponential fit; however, multiple methods exist, assuming constant or non-constant dose rate, bi- or monoexponential formula, as well as the other parameters which might vary. The third is related to the number of patients. Larger cohorts are necessary to validate such findings, particularly with regards to the complication rates. Moreover, our study does not answer the question on the optimal BED values for decreasing the toxicity, due to the small sample size and further the small number of events in this cohort. Another potential limitation is the choice of the alpha/beta ratio, which was considered 2.47, based upon previous studies [32], suggesting such value as the reference value for normal brain.

## Conclusion

The present study suggests a key role of BED for tremor arrest after thalamotomy for ET. An open question is whether modulating the BED values as function of cobalt-decay would allow to further increase the efficacy and decrease the toxicity of such technique.

A BED between 4300 and 4500 Gy_2.47_ and beam-on time of not more than 70 min are suggestive for better outcome.

Further studies are needed to determine how this increase in BED values and shorter treatment times contribute (or not) to toxicity to functional tissues and to evaluate whether further technical adjustments should be made.

## Data Availability

N/A.
